# 1,4-Bis[3-chloro-2-(chloro­meth­yl)prop­yl]benzene

**DOI:** 10.1107/S1600536809000609

**Published:** 2009-02-06

**Authors:** Haitao Xi, Yajun Gao, Xiaoqiang Sun, Zhijun Ma, Minqiu Xiong

**Affiliations:** a School of Chemistry and Chemical Engineering, Jiangsu Polytechnic University, Changzhou 213164, People’s Republic of China

## Abstract

The title mol­ecule, C_14_H_18_Cl_4_, possesses a crystallographically imposed inversion centre, which coincides with the centre of benzene ring. In the absence of classical inter­molecular inter­actions, van der Waals forces help the mol­ecules to pack in the crystal.

## Related literature

For related crystal structures, see: Chen *et al.* (2005 ▶); Gao *et al.* (2009 ▶). For general background, see Amabilino & Stoddart (1995 ▶).
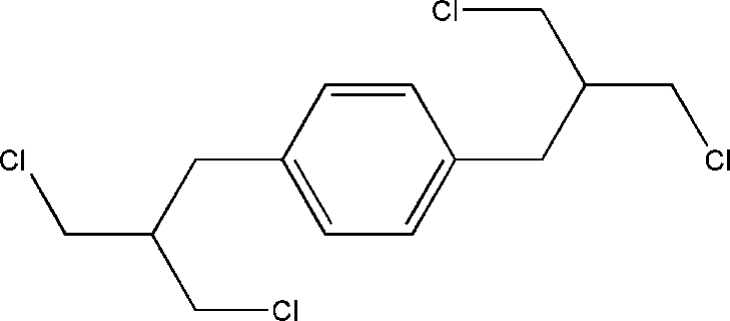


## Experimental

### 

#### Crystal data

C_14_H_18_Cl_4_
*M* *_r_* = 328.08
Monoclinic, 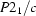

*a* = 6.518 (3) Å
*b* = 14.680 (6) Å
*c* = 8.433 (4) Å
β = 106.335 (5)°
*V* = 774.3 (6) Å^3^
*Z* = 2
Mo *K*α radiation
μ = 0.75 mm^−1^
*T* = 291 (2) K
0.30 × 0.26 × 0.24 mm

#### Data collection

Bruker SMART APEX CCD diffractometer
Absorption correction: multi-scan (*SADABS*; Bruker, 2000 ▶) *T*_min_ = 0.807, *T*_max_ = 0.842
4423 measured reflections
1679 independent reflections
1275 reflections with *I* > 2σ(*I*)
*R*_int_ = 0.066

#### Refinement

*R*[*F* ^2^ > 2σ(*F* ^2^)] = 0.040
*wR*(*F* ^2^) = 0.113
*S* = 1.09
1679 reflections
82 parameters
H-atom parameters constrained
Δρ_max_ = 0.32 e Å^−3^
Δρ_min_ = −0.32 e Å^−3^

### 

Data collection: *SMART* (Bruker, 2000 ▶); cell refinement: *SAINT* (Bruker, 2000 ▶); data reduction: *SAINT*; program(s) used to solve structure: *SHELXTL* (Sheldrick, 2008 ▶); program(s) used to refine structure: *SHELXTL*; molecular graphics: *SHELXTL*; software used to prepare material for publication: *SHELXTL*.

## Supplementary Material

Crystal structure: contains datablocks I, global. DOI: 10.1107/S1600536809000609/cv2502sup1.cif

Structure factors: contains datablocks I. DOI: 10.1107/S1600536809000609/cv2502Isup2.hkl

Additional supplementary materials:  crystallographic information; 3D view; checkCIF report

## Figures and Tables

**Fig. 1.**
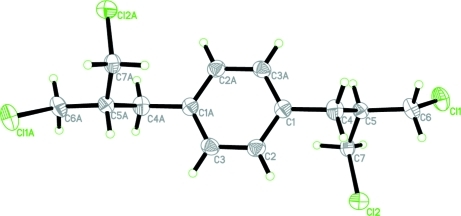
The molecular structure of (I) showing the atom-labelling scheme and 30% probability displacement ellipsoids [symmetry code: (A)-x, -y + 2, -z + 1].

## supplementary crystallographic information

### Comment

The molecular recognition between a π-electron-rich hydroquinone ring and π-electron-deficient cyclophane has provided the inspiration for the self-assembly of a large number of catenanes (Amabilino & Stoddart, 1995). In our study of the applications of fused bipyridine cyclophane compounds in the self-assembly of supramolecular systems, we obtained tetraethyl 2,2'-(*p*-phenylenedimethylene)dimalonate (Chen *et al.*, 2005), which was used in the synthesis of 2,2'-(*p*-phenylenedimethylene)bis(propane-1,3-diol) (Gao *et al.*, 2009). The title compound, (I),  was obtained by the chlorination of the diol. Herewith we present the crystal structure of (I) (Fig. 1).

### Experimental

The 2,2'-(p-phenylenedimethylene)bis(propane-1,3-diol), used in this study, was obtained in accordance with the Gao *et al.* (2005). In a flame-dryed, round-bottomed flask was placed SOCl_2_(5 mL) and *p*-C_6_H_4_[CH_2_CH(CH_2_OH)_2_]_2_(0.508 g,2 mmol) was slowly added under stirring. The mixture was heated up to 333 K. The solvent was evaporated and the resulting oil was chromatographed on a silica-gel column, yielding the title compound (0.51 g, 77%). M.p. 353–354 K.

### Refinement

All H atoms were geometrically positioned (C–H 0.93-0.98%A) and treated as riding, with  Uiso(H) = 1.2Ueq(C).

### Crystal data

**Table:**

C_14_H_18_Cl_4_	*F*(000) = 340
*M**_r_* = 328.08	*D*_x_ = 1.407 Mg m^−^^3^
Monoclinic, *P*2_1_/*c*	Mo *K*α radiation, λ = 0.71073 Å
Hall symbol: -P 2ybc	Cell parameters from 1607 reflections
*a* = 6.518 (3) Å	θ = 2.8–26.1°
*b* = 14.680 (6) Å	µ = 0.75 mm^−^^1^
*c* = 8.433 (4) Å	*T* = 291 K
β = 106.335 (5)°	Block, colourless
*V* = 774.3 (6) Å^3^	0.30 × 0.26 × 0.24 mm
*Z* = 2	

### Data collection

**Table:**

Bruker SMART APEX CCD diffractometer	1679 independent reflections
Radiation source: sealed tube	1275 reflections with *I* > 2σ(*I*)
graphite	*R*_int_ = 0.066
φ and ω scans	θ_max_ = 27.0°, θ_min_ = 2.8°
Absorption correction: multi-scan (*SADABS*; Bruker, 2000)	*h* = −8→7
*T*_min_ = 0.807, *T*_max_ = 0.842	*k* = −18→14
4423 measured reflections	*l* = −10→10

### Refinement

**Table:**

Refinement on *F*^2^	Primary atom site location: structure-invariant direct methods
Least-squares matrix: full	Secondary atom site location: difference Fourier map
*R*[*F*^2^ > 2σ(*F*^2^)] = 0.040	Hydrogen site location: inferred from neighbouring sites
*wR*(*F*^2^) = 0.113	H-atom parameters constrained
*S* = 1.09	*w* = 1/[σ^2^(*F*_o_^2^) + (0.0483*P*)^2^ + 0.0345*P*] where *P* = (*F*_o_^2^ + 2*F*_c_^2^)/3
1679 reflections	(Δ/σ)_max_ < 0.001
82 parameters	Δρ_max_ = 0.32 e Å^−^^3^
0 restraints	Δρ_min_ = −0.32 e Å^−^^3^

### Special details

**Table:**

Geometry. All e.s.d.'s (except the e.s.d. in the dihedral angle between two l.s. planes) are estimated using the full covariance matrix. The cell e.s.d.'s are taken into account individually in the estimation of e.s.d.'s in distances, angles and torsion angles; correlations between e.s.d.'s in cell parameters are only used when they are defined by crystal symmetry. An approximate (isotropic) treatment of cell e.s.d.'s is used for estimating e.s.d.'s involving l.s. planes.The structures were solved with direct methods and refined with full-matrix least-squares techniques using the *SHELXTL*. The coordinates of the non-hydrogen atoms were refined anisotropically, and the positions of the H atoms were positioned geometrically and refined using a riding model with C—H = 0.93–0.97 Å, and with *U*_iso_(H) = 1.2 times*U*_eq_(C).
Refinement. Refinement of *F*^2^ against ALL reflections. The weighted *R*-factor *wR* and goodness of fit *S* are based on *F*^2^, conventional *R*-factors *R* are based on *F*, with *F* set to zero for negative *F*^2^. The threshold expression of *F*^2^ > σ(*F*^2^) is used only for calculating *R*-factors(gt) *etc*. and is not relevant to the choice of reflections for refinement. *R*-factors based on *F*^2^ are statistically about twice as large as those based on *F*, and *R*- factors based on ALL data will be even larger.

### Fractional atomic coordinates and isotropic or equivalent isotropic displacement parameters (Å^2^)

**Table:**

	*x*	*y*	*z*	*U*_iso_*/*U*_eq_	
C1	0.1873 (3)	0.97482 (12)	0.6208 (2)	0.0415 (4)	
C2	0.0205 (4)	1.01687 (13)	0.6634 (2)	0.0482 (5)	
H2	0.0327	1.0284	0.7741	0.058*	
C3	−0.1637 (3)	1.04196 (13)	0.5447 (3)	0.0477 (5)	
H3	−0.2733	1.0705	0.5764	0.057*	
C4	0.3901 (3)	0.94730 (13)	0.7505 (3)	0.0489 (5)	
H7A	0.3999	0.9812	0.8511	0.059*	
H7B	0.5123	0.9638	0.7122	0.059*	
C5	0.4008 (3)	0.84467 (13)	0.7898 (2)	0.0410 (4)	
H8	0.3984	0.8123	0.6877	0.049*	
C6	0.6155 (3)	0.82501 (15)	0.9136 (3)	0.0543 (5)	
H9A	0.6174	0.8515	1.0194	0.065*	
H9B	0.7275	0.8539	0.8764	0.065*	
C7	0.2135 (3)	0.81085 (13)	0.8444 (2)	0.0444 (5)	
H10A	0.2273	0.7456	0.8629	0.053*	
H10B	0.0828	0.8218	0.7571	0.053*	
Cl1	0.67066 (10)	0.70596 (4)	0.94037 (8)	0.0736 (3)	
Cl2	0.19547 (9)	0.86582 (4)	1.03025 (7)	0.0635 (2)	

### Atomic displacement parameters (Å^2^)

**Table:**

	*U*^11^	*U*^22^	*U*^33^	*U*^12^	*U*^13^	*U*^23^
C1	0.0465 (11)	0.0298 (9)	0.0488 (11)	−0.0015 (8)	0.0141 (9)	0.0050 (8)
C2	0.0586 (13)	0.0437 (11)	0.0440 (11)	0.0046 (9)	0.0172 (10)	−0.0002 (8)
C3	0.0535 (12)	0.0389 (11)	0.0560 (12)	0.0063 (9)	0.0241 (10)	0.0036 (9)
C4	0.0424 (11)	0.0438 (11)	0.0570 (12)	−0.0062 (8)	0.0082 (9)	0.0026 (9)
C5	0.0389 (10)	0.0396 (10)	0.0443 (10)	0.0017 (8)	0.0114 (8)	−0.0017 (8)
C6	0.0368 (11)	0.0513 (12)	0.0718 (14)	0.0052 (9)	0.0101 (10)	0.0022 (10)
C7	0.0397 (11)	0.0414 (11)	0.0480 (11)	−0.0012 (8)	0.0058 (9)	0.0009 (8)
Cl1	0.0629 (4)	0.0603 (4)	0.0915 (5)	0.0227 (3)	0.0119 (3)	0.0086 (3)
Cl2	0.0614 (4)	0.0795 (4)	0.0538 (4)	0.0101 (3)	0.0228 (3)	0.0004 (3)

### Geometric parameters (Å, °)

**Table:**

C1—C3^i^	1.382 (3)	C5—C7	1.504 (3)
C1—C2	1.383 (3)	C5—C6	1.520 (3)
C1—C4	1.515 (3)	C5—H8	0.9800
C2—C3	1.380 (3)	C6—Cl1	1.786 (2)
C2—H2	0.9300	C6—H9A	0.9700
C3—C1^i^	1.382 (3)	C6—H9B	0.9700
C3—H3	0.9300	C7—Cl2	1.796 (2)
C4—C5	1.540 (3)	C7—H10A	0.9700
C4—H7A	0.9700	C7—H10B	0.9700
C4—H7B	0.9700		
			
C3^i^—C1—C2	118.01 (19)	C6—C5—C4	108.10 (16)
C3^i^—C1—C4	120.55 (19)	C7—C5—H8	107.2
C2—C1—C4	121.44 (18)	C6—C5—H8	107.2
C3—C2—C1	121.17 (19)	C4—C5—H8	107.2
C3—C2—H2	119.4	C5—C6—Cl1	112.74 (15)
C1—C2—H2	119.4	C5—C6—H9A	109.0
C2—C3—C1^i^	120.82 (19)	Cl1—C6—H9A	109.0
C2—C3—H3	119.6	C5—C6—H9B	109.0
C1^i^—C3—H3	119.6	Cl1—C6—H9B	109.0
C1—C4—C5	113.20 (15)	H9A—C6—H9B	107.8
C1—C4—H7A	108.9	C5—C7—Cl2	112.11 (13)
C5—C4—H7A	108.9	C5—C7—H10A	109.2
C1—C4—H7B	108.9	Cl2—C7—H10A	109.2
C5—C4—H7B	108.9	C5—C7—H10B	109.2
H7A—C4—H7B	107.8	Cl2—C7—H10B	109.2
C7—C5—C6	113.40 (16)	H10A—C7—H10B	107.9
C7—C5—C4	113.42 (15)		
			
C3^i^—C1—C2—C3	−0.4 (3)	C1—C4—C5—C6	176.91 (17)
C4—C1—C2—C3	179.85 (17)	C7—C5—C6—Cl1	64.6 (2)
C1—C2—C3—C1^i^	0.4 (3)	C4—C5—C6—Cl1	−168.74 (14)
C3^i^—C1—C4—C5	−77.5 (2)	C6—C5—C7—Cl2	62.71 (19)
C2—C1—C4—C5	102.2 (2)	C4—C5—C7—Cl2	−61.10 (19)
C1—C4—C5—C7	−56.4 (2)		

Symmetry codes: (i) −*x*, −*y*+2, −*z*+1.
